# External validity in healthy public policy: application of the RE-AIM tool to the field of housing improvement

**DOI:** 10.1186/1471-2458-12-633

**Published:** 2012-08-09

**Authors:** Hilary J Thomson, Sian Thomas

**Affiliations:** 1 MRC Social & Public Health Sciences Unit, Glasgow, Scotland, G12 8RZ, UK

**Keywords:** External validity, Healthy public policy, Research transfer, Socio-economic determinants of health

## Abstract

**Background:**

Researchers and publishers have called for improved reporting of external validity items and for testing of existing tools designed to assess reporting of items relevant to external validity. Few tools are available and most of this work has been done within the field of health promotion.

**Methods:**

We tested a tool assessing reporting of external validity items which was developed by Green & Glasgow on 39 studies assessing the health impacts of housing improvement. The tool was adapted to the topic area and criteria were developed to define the level of reporting, e.g. “some extent”. Each study was assessed by two reviewers.

**Results:**

The tool was applicable to the studies but some items required considerable editing to facilitate agreement between the two reviewers. Levels of reporting of the 17 external validity items were low (mean 6). The most commonly reported items related to outcomes. Details of the intervention were poorly reported. Study characteristics were not associated with variation in reporting.

**Conclusions:**

The Green & Glasgow tool was useful to assess reporting of external validity items but required tailoring to the topic area. In some public health evaluations the hypothesised impact is dependent on the intervention effecting change, e.g. improving socio-economic conditions. In such studies data confirming the function of the intervention may be as important as details of the components and implementation of the intervention.

## Background

Improving the use of research findings in policy and practice requires, among other things, clear reporting of external validity items [1-3]. External validity sometimes referred to as generalisability, means the extent to which causal inferences reported in one study can be applied to different populations, setting, treatments and outcomes [4,5]. For interventions this requires clear reporting of population characteristics (including setting and reach of the intervention), details of the intervention (including implementation, and adaptation to local settings), outcomes, and sustainability of the intervention and impacts [6,7]. Improved reporting of these items can assist the reader in judging the applicability and relevance of study findings to their own situation. It has been argued that improved reporting of external validity items may improve the usefulness and the appropriate use of research findings, as well as potentially contributing to improved quality of available evidence [2,3].

Despite an emerging acknowledgement by publishers of the importance of external validity, there is little clear guidance on what should be reported to facilitate judgements about external validity of study findings [6]. Within the health field much has been devoted to the development of tools to assess internal validity, but far less to external validity. One tool which articulates the required external validity items has been developed by the members of the RE-AIM team [8]. This tool is informed by Cronbach et al’s work on generalisability theory and the related UTOS elements [9]; Units (e.g. individual patients, moderator variables, sub-populations), Treatments (variations in treatment delivery or modality), Occasions (e.g. patterns of maintenance or relapse over time in response to treatments), and Settings (e,g, medical clinics, worksites, schools in which the intervention is being implemented and evaluated). The RE-AIM checklist was developed for public health interventions, specifically within the field of health promotion. While acknowledging the limitations of developing a standard tool the authors of this checklist have called for piloting to test and refine available tools [6]. The tool has been applied to studies of health promotion interventions [10,11], but to our knowledge, has not been tested in the field of healthy public policy, that is non-health sector interventions such as education, welfare, housing, transport etc.

We used a recent systematic review of the health impacts of housing improvements [12] to investigate levels of reporting of external validity items using the RE-AIM tool [8]. This brief report presents the level of reporting and reflects on how reporting of external validity might need to be adapted for use in the broader field of healthy public policy.

## Methods

The Green & Glasgow tool was tailored to meet the characteristics of the studies being assessed. This required some rewording of the original questions to improve clarity or adapting some details of the original questions developed by Green & Glasgow to be more appropriate to a group of housing studies. A set of criteria to assess the extent to which each external validity item had been reported was developed by both authors, i.e. “large extent”, “some extent”, “unclear”, “not at all” or “not applicable (N/A)”. A summary of the items assessed is provided in Table 1. The full version of the tool with the criteria for assessment is available as Additional file 1.

**Table 1:** Reworded external validity items and extent of reporting by item (n = 39 studies) *

		**Large extent**	**Some extent**	**Unclear**	**Not at all**
**A Population: Representativeness of target population, setting & reach of intervention**					
1	Are data presented on variations in participation rate in improved housing interventions by a) setting b) delivery staff/organisations c) residents (for intervention among general target population not study area)	0	0	0	39
2	Is the intended target audience for adoption clearly described	11	18	8	2
3	Is the intended target setting for adoption clearly described?	4	27	5	3
4	Is there analysis of the baseline socio-demographic and ‘condition tested’ (health status) of evaluation participants versus non-participants? (relating to evaluation population only)	0	0	2	37
**B Intervention: Implementation & adaptation**					
5	Are data presented on consistency of implementation of intervention & its different components?	0	2	2	35
6	Are data presented on the level of training of experience required to deliver the programme or quality of implementation by different types of staff?	0	1	1	37
7	Is information reported on whether/how the intervention is modified to individuals/households within the study?	5	6	0	11
8	Are data presented on mediating factors or processes (mechanisms) through which the intervention had an impact?	2	12	4	21
**C Outcomes for decision making**					
9	Are the reported health (even if only one measure of health is comparable) outcomes comparable to wider policy/other studies?	23	14	0	2
10	Have additional outcomes of potential adverse impacts been reported? e.g. socio-economic impacts	4	21	1	13
11	Have authors demonstrated consideration of variation in reported health outcomes (key outcome of interest) by population sub-groups, or intervention setting/delivery staff?	2	4	1	32
12	Is there sensitivity analysis of dose–response/threshold level required to observe health effect (effect on key outcome of interest not proxies)?	3	4	1	31
13	Are data on costs presented? Are standard economic/accounting methods used?	2	19	0	18
**D Maintenance and institutionalisation of intervention**					
14	Are long term effects reported? (12 months or longer since exposure to the intervention)	10	13	4	11
15	Are data reported on the sustainability (or reinvention or evolution) of programme implementation and intervention, at least 12 months after the formal evaluation?	0	0	0	29
16 a	Is the drop-out rate/attrition reported?	19 (Yes)		10 (N/A)	
16 b	Are data on attrition by baseline health status of dropouts reported and are analyses conducted of the representativeness of remaining sample at time of final follow-up (or main follow-up time point- as appropriate)?	0	0	0	29 (10 N/A)

(adapted from Green LW, Glasgow RE. Evaluating the Relevance, Generalization, and Applicability of Research: Issues in External Validation and Translation Methodology. *Eval Health Prof* 2006;29(**1**):126–153.)

* see Additional file 1 for full details of external validity assessment tool.

Thirty nine intervention studies which had assessed the health impacts of housing improvement and were included in an earlier systematic review [12] were assessed independently by two reviewers for the extent of reporting of the external validity items detailed in the tool, i.e. “large extent”, “some extent”, “unclear”, “not at all” or “not applicable (N/A)”. Disagreements on the assessment between the two reviewers were resolved by discussion and where disagreements persisted the questions or assessment criteria (large extent, some extent etc) were further clarified. Only the studies and related papers included in the published review were included in this assessment, this included the key paper for each study (n = 39) plus a further 29 publications linked with the included studies. Authors were not contacted to obtain further information on external validity items.

Data were extracted and entered onto a Microsoft Access database ©. A summary score for the level of reporting was calculated for each domain (Reach; Implementation; Outcomes; Maintenance). The codes for each item were converted into a numeric value (“large extent” or “some extent” = 1 “unclear” or “not at all” or “N/A” = 0). A sub-total score for each domain and a total score was calculated for each study. The score indicating the level of reporting for each of the external validity items, and summary scores for the domains were tabulated along with key study characteristics identified in the original systematic review, these included intervention type, context, study design, and overall assessment of internal validity (for details of the internal validity assessment and definitions of the study intervention categories see the full systematic review [12]).

## Results

### Application of Green & Glasgow tool

There was considerable disagreement between the two reviewers requiring substantial iteration to clarify the meaning and purpose of some of the external validity items (Table 1 & Additional file 1). Three items were particularly difficult to clarify (2, 3 & 11) and were reworked to relate to descriptions of the study population or setting and eligibility for the intervention. Eight items were rephrased for clarification and/or inclusion of terms or issues relevant to the field. Item 16 was split into two. Five items (5, 6, 13, 14, & 15) remained unchanged from the original tool developed by Green & Glasgow [8]. Following these edits there was improved agreement between the reviewers but some disagreements persisted and were resolved by discussion. The two items with greatest disagreement were 2 & 3 where half or more of the assessments differed (50% and 68.8% respectively). Levels of agreement were highest for items 5, 6, 15 and 16.

### Reporting of external validity in housing improvement studies

Reporting of external validity items was low across the studies (Table 2 & Additional file 2); overall 35.3 % of items were reported (mean 6, range 2–9, median 6). Within each external validity domain (Reach, Implementation, Outcomes, & Maintenance) few studies reported more than half the items either “to some extent” or “to a large extent”. The “outcomes” domain had a greater number of reported items among the studies (mean items reported 49.8%); the “intervention” domain was the most poorly reported (mean items reported 29.0%). No item was universally reported. Item 9 & 16a were most commonly reported. Three items were not reported in any study: items 1, 14, and 15 (Table 2). There was little variation in the number of reported items between intervention type, location or date of study. The better quality studies reported more external validity items (mean number of external validity items reported by Internal Validity Grade A/B/C 6.7/5.36/5.4).

**Table 2:** Number of external validity items reported in each study by domain

**Author year**	**Study design**	**Internal validity grade**	**External validity domains (maximum possible score)**				
			**Reach & representat’n (4)**	**Implementat’n & adaptation (4)**	**Outcomes (5)**	**Maintenance & institutionalisat’n (4)**	**Total (17)**
**Intervention: Warmth & Energy Efficiency improvements (post 1980) (n = 19)**							
Heyman et al. 2010	RCT	A	1	2	3	2	8
Braubach et al. 2008	CBA	A	2	1	1	1	5
Howden-Chapman et al. 2008	RCT	A	2	1	1	1	5
Barton et al. 2007	RCT	A	2	1	3	2	8
Howden-Chapman et al. 2007	RCT	A	2	0	3	1	6
Platt et al. 2007	CBA	A	1	1	4	2	8
Lloyd et al. 2008	CBA	B	1	0	3	2	6
Shortt et al. 2007	CBA	B	2	2	2	2	8
Somerville et al. 2000	UBA	B	2	1	3	1	7
Hopton et al. 1996	CBA	B	2	0	2	1	5
Warm Front Study Group 2006	RC	C	1	1	5	0	7
Allen 2005 a	UBA	C	1	1	2	2	6
Allen 2005 b	UBA	C	1	2	1	1	5
Health Action Kirklees 2005	R	C	1	0	1	0	2
Eick et al. 2004	RCT	C	2	1	4	2	9
Winder et al. 2003	UBA	C	1	0	0	1	2
Caldwell et al. 2001	CBA	C	2	2	3	2	9
Green et al. 1999	RC	C	1	0	3	0	4
Iversen et al. 1986	CBA	C	1	0	1	1	3
**Mean (range)**			1.47 (1–2)	0.84 (0–2)	2.37 (0–4)	1.26 (0–2)	5.95 (2–9)
**Intervention: Rehousing/retrofitting +/− neighbourhood renewal (post 1995) (n = 10)**							
Kearns et al. 2008	CBA	A	1	1	3	2	7
Thomson et al. 2007	CBA	A	2	0	2	2	6
Critchley et al. 2004	CBA	A	0	1	4	1	6
Thomas et al. 2005	CBA	B	2	1	3	1	7
Barnes et al. 2003	CBA	B	2	0	2	2	6
Evans et al. 2002	CBA	B	1	0	2	0	3
Blackman et al. 2001	UBA	C	2	0	2	2	6
Wells 2000	UBA	C	2	0	1	2	5
Ambrose 1999	UBA	C	2	1	3	2	8
Halpern 1995	XUBA	C	2	1	2	1	6
**Mean (range)**			1.6 (0–2)	0.50 (0–1)	2.40 (1–4)	1.50 (0–2)	6 (3–8)
**Intervention: Provision of basic housing needs/developing country intervention (n = 6)**							
Cattaneo et al. 2006	RC	B	2	1	4	1	8
Choudhary et al. 2002	RC	B	1	1	3	1	6
Aga Khan Health Service 2001	XCBA	B	1	1	2	1	5
Spiegel et al. 2003	XCBA	C	2	0	1	1	4
Aiga et al. 2002	XCBA	C	1	1	3	0	5
Wolff et al. 2001	XCBA	C	2	0	2	1	5
**Mean (range)**			1.50 (1–2)	0.67 (0–1)	2.67 (1–4)	0.83 (0–1)	5.5 (4–8)
**Intervention: Rehousing from slums (pre 1965) (n = 4)**							
Wilner et al. 1960	CBA	A	2	1	3	2	8
McGonigle et al. 1936	XCBA	B	2	1	4	1	8
Ferguson 1954	RC	C	1	0	1	1	3
Chapin 1938	UBA	C	2	1	4	2	9
**Mean (range)**			1.75 (1–2)	0.75 (0–1)	3.00 (2–4)	1.50 (1–2)	7.00 (3–9)
**TOTAL (n = 39 studies) MEAN (range)**			1.54 (0–2)	0.72 (0–2)	2.49 (0–4)	1.28 (0–2)	6.00 (2–9)

Study design: RCT: Randomised Controlled Trial; CBA: Controlled Before & After; UBA: Uncontrolled Before & After; XCBA: Cross-sectional Controlled Before & After; XUBA: Cross-sectional Uncontrolled Before & After; RC: Retrospective controlled; R: Retrospective uncontrolled.

List of references available in reference 4 or from the author.

## Discussion

Following adaptation and development of detailed assessment criteria relevant to the studies being assessed, the external validity tool was successfully applied to studies of housing improvement drawing on the primary paper and associated papers available at the time of the original review [12]. Reporting of external validity items was low overall (median 35.6%) and across individual domains in the tool. This is comparable to the level of reporting in a group of studies of childhood obesity prevention (median 34.5%) [10].

The studies we assessed represented a broad range of study designs, contexts, and other aspects of study quality and interventions, as well as representing both published and unpublished studies. There was no suggestion of a link between study characteristics and reporting of external validity. The apparent link between internal and external validity reporting may be explained by the overlap in assessed items, specifically attrition and sample selection. It is possible that author contact and publications since the original searches in 2008 will have reported additional relevant data.

The domain relating to the intervention (items 5–8) was least often reported; Klesges et al. reported a similar issue [10]. While faithful replication of a novel intervention may depend on detailed reporting of intervention components and implementation [13], this may be less important for a well established intervention, such as housing improvements. Moreover, for complex social interventions, such as housing improvements, data confirming intervention function may be of more value than the details of intervention form [14]. Data on changes effected by the intervention, such as improved warmth, may be used to refine generalisable theories regarding tackling socio-economic determinants of health even where the specific intervention may not be widely generalisable. Where there is evidence to support the theory that changes in an intermediate outcome can lead to health, selection and implementation of appropriate and effective interventions to improve a named socio-economic outcome, such as warmth, may be made locally. This issue is of particular relevance to interventions where the hypothesised health impacts are dependent on the intervention affecting an intermediate variable, for example healthy public policy interventions tackling socio-economic determinants of health.

There is little doubt that reporting of external validity items needs improving. However, in agreement with Green et al, development of a standard tool may not be appropriate [6]. In our study there was poor agreement between the two assessors in the interpretation of the tool. In response it was necessary to amend and clarify meaning to allow appropriate application of the tool to this group of studies. Specifically, aspects of maintenance and reach require tailoring to the intervention, and reporting of differential effects using sub-group analysis will inevitably be limited where studies are typically small*.* Where a well established intervention like housing improvement is being evaluated items assessing reporting of population details (namely items 2 & 3) may require editing to clarify whether they relate to reporting of the target population and context for the study or the wider intervention. We chose to focus on the study population. In addition, criteria to indicate the extent of reporting were developed to reflect issues pertinent to our particular group of studies. The use of graded criteria improves the sensitivity and interpretation of the tool beyond the previous version which is restricted to a binary assessment [10,11].

## Conclusion

The RE-AIM checklist provides a useful framework to guide authors about which external validity items to report but, as indicated in earlier discussions, strict adherence to the checklist may not be appropriate. Despite this there is a need for authors to improve reporting of items to improve the transferability of the research findings. Within health public policy, the hypothesised health impacts of interventions are often dependent on an intermediate impact on a socio-economic determinant of health. Data confirming the function of the intervention may be as important as details of the intervention form and may help refine generalisable theories about the health impacts of tackling socio-economic determinants of health.

## Competing interests

HT & ST are not aware of any competing interest in this work.

## Authors’ contributions

HT & ST selected and assessed each study. HT prepared and analysed the data and wrote the paper with comments from ST. Both authors read and approved the final manuscript.

## Funding

HT & ST are funded by the Chief Scientist Office at the Scottish Government Health Directorate as part of the Evaluating Social Interventions programme at the MRC Social and Public Health Science Unit, (U.130059812).

## Pre-publication history

The pre-publication history for this paper can be accessed here:

http://www.biomedcentral.com/1471-2458/12/633/prepub

## Supplementary Material

Additional file 1 External validity reporting assessment tool: amended version and criteria developed by Thomson & Thomas. Click here for file

Additional file 2 Assessment codes for individual external validity items with domain score sub-totals (ST) and total score by study. Click here for file

## References

[B1] Bonell C Oakley A Hargreaves J Strange V Rees R Assessment of generalisability in trials of health interventions: suggested framework and systematic review BMJ 2006 333 7563 346 349 10.1136/bmj.333.7563.346 16902217 PMC1539056

[B2] Burchett H Umoquit M Dobrow M How do we know when research from one setting can be ueful in another? A review of external validity, applicability and tranferability frameworks J Health Serv Res Policy 2011 16 4 238 244 10.1258/jhsrp.2011.010124 21965426

[B3] Glasgow RE Green L Klesges LM Abrams D Fisher E Goldstein M Hayman L Ockene J Orleans C External validity: we need to do more Ann Behav Med 2006 31 105 108 10.1207/s15324796abm3102_1 16542124

[B4] Glasgow RE Green LW Ammerman A A focus on external validity Eval Health Prof 2007 30 2 115 117 10.1177/0163278707300627

[B5] Shadish WR Cook TD Campbell DT Experimental and quasi-experimental designs for generalized causal inference 2002 Boston: Houghton Mifflin Company

[B6] Green LW Glasgow RE Atkins D Stange K Making Evidence from Research More Relevant, Useful, and Actionable in Policy, Program Planning, and Practice: Slips "Twixt Cup and Lip" Am J Prev Med 2009 37 6, Supplement 1 S187 S191 10.1016/j.amepre.2009.08.017 19896017

[B7] Steckler A McLeroy KR The Importance of External Validity Am J Public Health 2008 98 1 9 10 10.2105/AJPH.2007.126847 18048772 PMC2156062

[B8] Green LW Glasgow RE Evaluating the Relevance, Generalization, and Applicability of Research: Issues in External Validation and Translation Methodology Eval Health Prof 2006 29 1 126 153 10.1177/0163278705284445 16510882

[B9] Cronbach LH Glesser GC Nanda H Rajaratnam N The dependability of behavioral measurements: Theory of generalizability for scores and profiles 1972 New York: John Wiley

[B10] Klesges LM Dzewaltowski DA Glasgow RE Review of External Validity Reporting in Childhood Obesity Prevention Research Am J Prev Med 2008 34 3 216 223 10.1016/j.amepre.2007.11.019 18312810

[B11] Klesges LM Williams NA Davis KS Buscemi J Kitzmann KM External Validity Reporting in Behavioral Treatment of Childhood Obesity: A Systematic Review Am J Prev Med 2012 42 2 185 192 10.1016/j.amepre.2011.10.014 22261216 PMC4573550

[B12] Thomson H Thomas S Sellstrom E Petticrew M The Health Impacts of Housing Improvement: A Systematic Review of Intervention Studies From 1887 to 2007 Am J Public Health 2009 99 S3 S681 S692 10.2105/AJPH.2008.143909 19890174 PMC2774202

[B13] Glasziou P Meats E Heneghan C Shepperd S What is missing from descriptions of treatment in trials and reviews? BMJ 2008 336 7659 1472 1474 10.1136/bmj.39590.732037.47 18583680 PMC2440840

[B14] Hawe P Shiell A Riley T Complex interventions: how “out of control” can a randomised controlled trial be? BMJ 2004 328 7455 1561 1563 10.1136/bmj.328.7455.1561 15217878 PMC437159

