# Supplementary material for: External validity in healthy public policy: application of the RE-AIM tool to the field of housing improvement
Source: BMC Public Health. 2012 Aug 9;12:633. doi: 10.1186/1471-2458-12-633 (PMC3481477; doi:10.1186/1471-2458-12-633)
Supplement: Additional file 1 — External validity reporting assessment tool: amended version and criteria developed by Thomson & Thomas. [file 1471-2458-12-633-S1.doc]

***Additional file 1: External validity reporting assessment tool: amended version and criteria developed by Thomson & Thomas***

| **A Population: Representativeness of target population, setting & reach of intervention** | | **Criteria for Large extent/Some extent/Not at all/Unclear** |
| --- | --- | --- |
| 1 | *Original from Green & Glasgow 2006*  Participation: Are there analyses of the participation rate among potential (a) settings, (b) delivery staff, and (c) patients (consumers)? | |
|  | Are data presented on variations in participation rate in improved housing interventions by a) setting b) delivery staff/organisations c) residents (for intervention among general target population not study area)  Clarification/criteria: Statement of variation in and influences on uptake of the intervention among the wider target population not just study sample/area. | Large extent: detailed data of key influences on uptake of intervention- considering characteristics of a) settings b) delivery staff/organisation c) residents  Some extent: some data on key influences on uptake provided  Unclear: unclear data on key influences on uptake provided  Not at all: no data on key influences on uptake provided |
| 2 | *Original from Green & Glasgow 2006*  Target audience: Is the intended target audience stated for adoption (at the intended settings such as worksites, medical offices, etc.) and application (at the individual level)? | |
|  | Is the intended target audience for adoption clearly described? (see 2 a & 2 b below)  2 a Are the study population (actual sample not study target population) clearly described?  2 b Is the eligibility of individuals or households to receive the intervention clearly described? | Large extent: study population AND eligibility clearly described (Yes 2 a & b)  Some extent: study population OR eligibility clearly described (Yes 2a OR 2b)  Unclear: unclear description of study population or eligibility  Not at all: no description of study population or eligibility (No 2a & 2b)  Yes/No  Yes/No |
| 3 | *Original from Green & Glasgow 2006*  Representativeness- Settings: Are comparisons made of the similarity of the settings in the study to the intended target audience of the program settings- or to those settings that decline to participate? | |
|  | Is the intended target setting for adoption clearly described? (see 3 a & 3 b below)  3 a Are the eligibility for the settings to receive the intervention clearly described?  3 b Is the study setting(s) clearly described? | Large extent: study setting AND eligibility clearly described (Yes 3a & 3B)  Some extent: study setting OR eligibility clearly described (Yes 3a OR 3B)  Unclear: unclear description of study setting or eligibility  Not at all: no description of study setting or eligibility (No 3a & 3b)  Yes/No N/A- for individual level interventions  Yes/No |

| 4 | *Original from Green & Glasgow 2006*  Representativeness- Individuals: Are analyses conducted of the similarity and differences between patients, consumers, or other subjects who participate versus either those who decline, or the intended target audience? | |
| --- | --- | --- |
|  | Is there analysis of the baseline socio-demographic and outcome (health) of evaluation participants versus non-participants? (relating to evaluation population only)  Clarification/criteria: Extent of comparison of baseline outcome measures (health status, housing conditions) between evaluation participants versus those who decline evaluation participation or others who have received the intervention but are not in the evaluation. | Large extent: data reported comparing of baseline health AND housing condition OR baseline health AND at least two socio-demographic variables (gender, age, socio-economic status, tenure) of evaluation participants and non-participants in study target area/population  Some extent: data comparing of one of the above outcomes;  Unclear: narrative describing above comparison  Not at all: no reference to comparison of representativeness of evaluation participants with respect to study target area/population |
| **B Intervention: Implementation & adaptation** | |  |
| 5 | *Original from Green & Glasgow 2006*  Consistent implementation: Unchanged | |
|  | Are data presented on consistency of implementation of different program components?  Clarification/criteria: Data on variation in intervention due to implementation (not due to tailoring to setting or individual need). This may include details of higher level policy contextual influence or importance of inter-agency working and partnership on implementation of intervention which may vary by setting- e.g. different local authority etc | Large extent: data reported across whole sample including stakeholder perceptions of influences  Some extent: data presented for sub-sample or detailed author narrative of perceived influences  Unclear: some indication of variation but no data, e.g. limited narrative  Not at all: not reported |
| 6 | *Original from Green & Glasgow 2006*  Staff expertise: Unchanged | |
|  | Are data presented on the level of training of experience required to deliver the programme or quality of implementation by different types of staff?  Clarification/criteria: Any data or reports of training of staff with respect to intervention implementation | Large extent: detailed reports of staffing and training influences on intervention  Some extent: some reports of staffing and training influences on intervention  Unclear: unclear reports of staffing and training influences on intervention  Not at all: no reports of staffing and training influences on intervention |
| 7 | *Original from Green & Glasgow 2006*  Program adaptation: Is information reported on the extent to which different settings modified or adapted the program to fit their setting? | |
|  | Is information reported on whether and how the intervention is modified to individuals/ households within the study?  Clarification/criteria: Extent to which housing improvement tailored to individual need | Large extent: detailed data on modification of intervention across study sample Some extent: some data on modification for some of the sample (would include where numbers of sample getting each intervention provided)  Unclear: indication that intervention is modified but no indication of proportion of sample that received the different interventions or clear data or on extent of variation  Not at all: no report of heterogeneity in intervention across study sample |
| 8 | *Original from Green & Glasgow 2006*  Mechanisms: Are data reported on the process(es) or mediating variables through which the program or policy achieved its effects? | |
|  | Are data presented on mediating factors or processes (mechanisms) through which the intervention had an impact?  Clarification/criteria: Mediating influence of characteristic of intervention on health, for example socio-economic impacts associated with housing intervention- *not mediating impacts on intervention*. | Large extent: qualitative/quantitative data on reported mediator reported for whole or part of sample and analysis to act as confirmation of mediator as important; Some extent: some indicative qualitative/quantitative data reported for all or part of sample suggesting influence of identified mediator  Unclear: only author speculation on mediating influences  Not at all: no data or reference to mediating factors |
| **C Outcomes for decision making** | |  |
| 9 | *Original from Green & Glasgow 2006*  Significant: Are outcomes reported in a way that can be compared to either clinical guidelines or public health goals? | |
|  | Are the reported health (even if only one measure of health is comparable) outcomes comparable to wider policy/other studies?  Clarification/criteria: Commonly used health outcomes assessed using existing metric, i.e. objective health outcome measure used- validated measures such as GHQ, HADS, SF-36, or self-reported health (not other self-reported symptoms such as asthma), or routine morbidity or mortality data gathered from census of health records e.g. clinic attendance for diarrhoea (not health service use). | Large extent: commonly used objective health measure used as key outcome  Some extent: direct measure of health used but not validated or commonly used  Unclear: unclear how comparable and widely used key outcome is  Not at all: key outcome is unique to study |
| 10 | *Original from Green & Glasgow 2006*  Adverse consequences: Do the outcomes reported include quality of life or potential negative outcomes? | |
|  | Have additional outcomes of potential adverse impacts been reported?  Clarification/criteria: Reporting of additional socio-economic outcomes associated with the intervention | Large extent: qualitative or quantitative data on socio-economic outcomes for whole study sample  Some extent: qualitative or quantitative data on socio-economic outcomes for sub-sample  Unclear: some additional data reported but unclear  Not at all: no additional outcomes reported or indication of adverse impacts |
| 11 | *Original from Green & Glasgow 2006*  Moderators: Are there any analyses of moderator effects- including of different sub-groups of participants and types of intervention staff- to assess robustness versus specificity of effects? | |
|  | Have authors demonstrated consideration of variation in reported health outcomes (key outcome of interest) by population sub-groups, or intervention setting/delivery staff?  Clarification/criteria:: Sub-group analysis by population sub-group/intervention setting or other factors related implementation of the intervention. Doesn’t include adjustment for sample demographics. | Large extent: adjustment for intervention variation in reported analysis for both population and intervention factors  Some extent: adjustment for intervention variation in reported analysis for population OR intervention factors  Unclear: author speculation about relationship between sub-group or intervention factors on outcomes  Not at all: no relevant sub-group analysis |
| 12 | *Original from Green & Glasgow 2006*  Sensitivity: Are there any sensitivity analysis of dose-response effects, threshold level or point of diminishing returns on the resources expended? | |
|  | Is there sensitivity analysis of dose-response effects, or threshold level required to observe a health effect (effect on key outcome of interest not proxies)?  Clarification/criteria: Analysis by extent of change in housing condition/intervention delivered | Large extent: sensitivity/sub-group analysis clearly reported for whole sample  Some extent: sensitivity/sub-group analysis reported for part of sample  Unclear: author speculation about possible dose-response effect/unclear sub-group analysis  Not at all: no relevant sub-group analysis |
| 13 | *Original from Green & Glasgow 2006*  Costs: Unchanged | |
|  | Are data on costs presented? Are standard economic/accounting methods used to fully account for costs?  Clarification/criteria: Economic analysis or cost data reported. | Large extent: health economic analysis  Some extent: cost data only (may include costs of intervention/programme or costs to residents)  Unclear: some data reported but unclear  Not at all: no data reported |
| **D Maintenance and institutionalisation of intervention** | |  |
| 14 | *Original from Green & Glasgow 2006*  Long term effects: Unchanged | |
|  | Are long term effects reported? (12 months or longer since exposure to the intervention)    Clarification/criteria: Time since initial exposure to intervention at final follow-up. | Large extent: 12 months or more for >50% of original sample  Some extent: 12 months or more for <50% original sample  Unclear: some data at 12 months is reported but unclear  Not at all: no data at 12 months or more |
| 15 | *Original from Green & Glasgow 2006*  Institutionalization: Unchanged | |
|  | Are data reported on the sustainability (or reinvention or evolution) of programme implementation and intervention, at least 12 months after the formal evaluation?  Clarification/criteria: Reports of sustained level of exposure to improved housing among movers 12 months after evaluation end. | Large extent- detailed reporting of sustained intervention exposure i.e. whether residents still living in improved housing AND whether improved housing conditions sustained at least 12 months after end of evaluation  Some extent: some reporting either whether residents still in improved housing OR housing conditions sustained  Unclear: some data reported but unclear  Not at all: no data on sustainability of the intervention reported |
| 16 | *Original from Green & Glasgow 2006*  Attrition: Are data on attrition by condition reported, and are analyses conducted of the representativeness of those who drop out? | |
| 16 a | Are withdrawals since baseline reported? | Yes/No N/A for retrospective studies |
| 16 b | Are data on attrition by baseline health status of dropouts reported and are analyses conducted of the representativeness of remaining sample at time of final follow-up (or main follow-up time point- as appropriate)?  Clarification/criteria: Include assessment of attrition, details of drop-outs reported, and accompanying analysis. | Large extent: data on baseline health status of drop-outs reported & compared with baseline health status of final sample  Some extent: data on baseline health status of drop-outs reported (drop-out numbers alone are not sufficient)  Unclear: some indication of baseline health status for drop-outs but no clear data n/a for retrospective studies gathering data at one timepoint.  Not at all: no data on baseline health data of drop-outs reported |
