# Supplementary material for: External validity in healthy public policy: application of the RE-AIM tool to the field of housing improvement
Source: BMC Public Health. 2012 Aug 9;12:633. doi: 10.1186/1471-2458-12-633 (PMC3481477; doi:10.1186/1471-2458-12-633)
Supplement: Additional file 2 — Assessment codes for individual external validity items with domain score sub-totals (ST) and total score by study. [file 1471-2458-12-633-S2.docx]

**Additional file 2: Assessment codes for individual external validity items with domain score sub-totals (ST) and total score by study**

| **Author & Year** | **Study design** | **IVG** | **Reach & representation** | | | | | **Implementation & adaptation** | | | | | **Outcomes** | | | | | | **Maintenance & institutionalisation** | | | | |  |  |
| --- | --- | --- | --- | --- | --- | --- | --- | --- | --- | --- | --- | --- | --- | --- | --- | --- | --- | --- | --- | --- | --- | --- | --- | --- | --- |
| **Item number** | | | **1** | **2** | **3** | **4** | **ST** | **5** | **6** | **7** | **8** | **ST** | **9** | **10** | **11** | **12** | **13** | **ST** | **14** | **15** | **16a** | **16b** | **ST** |  | **Total** |
| **Intervention: Warmth & Energy Efficiency improvements (post 1980) (n=19)** | | | | | | | | | | | | | | | | | | | | | | | | | |
| Heyman et al 2010 | RCT | A | N | L | U | N | 1 | N | N | S | S | 2 | S | S | N | N | S | 3 | L | N | Yes | N | 2 |  | 8 |
| Braubach et al 2008 | CBA | A | N | S | S | N | 2 | N | N | S | N | 1 | L | N | N | N | N | 1 | N | N | Yes | N | 1 |  | 5 |
| Howden-Chapman et al 2008 | RCT | A | N | L | S | N | 2 | N | N | S | N | 1 | L | N | N | N | N | 1 | N | N | Yes | N | 1 |  | 5 |
| Barton et al 2007 | RCT | A | N | L | S | N | 2 | N | N | U | S | 1 | S | S | N | N | L | 3 | L | N | Yes | N | 2 |  | 8 |
| Howden-Chapman et al 2007 | RCT | A | N | L | S | N | 2 | U | U | U | N | 0 | L | S | N | N | L | 3 | N | N | Yes | N | 1 |  | 6 |
| Platt et al 2007 | CBA | A | N | L | N | N | 1 | N | N | U | S | 1 | L | S | L | N | S | 4 | L | N | Yes | N | 2 |  | 8 |
| Lloyd et al 2008 | CBA | B | N | N | S | N | 1 | N | N | N | N | 0 | L | S | N | N | S | 3 | L | N | Yes | N | 2 |  | 6 |
| Shortt et al 2007 | CBA | B | N | S | L | N | 2 | N | S | S | N | 2 | L | S | N | U | N | 2 | L | N | Yes | N | 2 |  | 8 |
| Somerville et al 2000 | UBA | B | N | L | L | N | 2 | N | N | S | N | 1 | L | S | N | N | S | 3 | N | N | Yes | N | 1 |  | 7 |
| Hopton et al 1996 | CBA | B | N | S | S | N | 2 | N | N | N | N | 0 | S | S | N | N | N | 2 | N | N | Yes | N | 1 |  | 5 |
| Warm Front Study Group 2006 | RC | C | N | N | S | N | 1 | N | N | L | N | 1 | L | S | S | L | S | 5 | N | N | na | na | 0 |  | 7 |
| Allen 2005 a | UBA | C | N | L | U | N | 1 | S | N | U | N | 1 | L | U | N | N | S | 2 | S | N | Yes | N | 2 |  | 6 |
| Allen 2005 b | UBA | C | N | L | U | N | 1 | S | N | S | N | 2 | L | N | N | N | N | 1 | N | N | Yes | N | 1 |  | 5 |
| Health Action Kirklees 2005 | R | C | N | S | N | N | 1 | U | N | U | N | 0 | L | N | N | N | N | 1 | N | N | na | na | 0 |  | 2 |
| Eick et al 2004 | RCT | C | N | L | S | N | 2 | N | N | L | U | 1 | S | S | N | S | S | 4 | S | N | Yes | N | 2 |  | 9 |
| Winder et al 2003 | UBA | C | N | L | U | N | 1 | N | N | U | N | 0 | N | N | N | N | N | 0 | N | N | Yes | N | 1 |  | 2 |
| Caldwell et al 2001 | CBA | C | N | S | S | N | 2 | N | N | L | S | 2 | S | S | N | N | S | 3 | S | N | Yes | N | 2 |  | 9 |
| Green et al 1999 | RC | C | N | U | S | N | 1 | N | N | N | N | 0 | S | S | N | N | S | 3 | U | N | na | na | 0 |  | 4 |
| Iversen et al 1986 | CBA | C | N | N | S | N | 1 | N | N | N | N | 0 | S | N | U | N | N | 1 | N | N | Yes | N | 1 |  | 3 |
|  |  |  |  |  |  |  |  |  |  |  |  |  |  |  |  |  |  |  |  |  |  |  |  |  |  |
| **Mean**  **(range)** |  |  |  |  |  |  | 1.47  (1-2) | |  |  |  | 0.84 (0-2) |  |  |  |  |  | 2.37 (0-4) |  |  |  |  | 1.26 (0-2) |  | 5.95 (2-9) |
| **Intervention: Rehousing/retrofitting +/- neighbourhood renewal (post 1995) (n=10)** | | | | | | | | | | | | | | | | | | | | | | | | | |
| Kearns et al 2008 | CBA | A | N | S | N | N | 1 | N | N | N | S | 1 | L | L | N | N | S | 3 | L | N | Yes | N | 2 |  | 7 |
| Thomson et al 2007 | CBA | A | N | S | S | N | 2 | N | N | U | U | 0 | L | S | N | N | N | 2 | L | N | Yes | N | 2 |  | 6 |
| Critchley et al 2004 | CBA | A | N | U | U | N | 0 | N | N | U | S | 1 | S | S | N | S | S | 4 | U | N | Yes | N | 1 |  | 6 |
| Thomas et al 2005 | CBA | B | N | S | S | U | 2 | N | N | L | U | 1 | L | N | N | L | S | 3 | U | N | Yes | N | 1 |  | 7 |
| Barnes et al 2003 | CBA | B | N | S | S | N | 2 | N | N | U | N | 0 | L | S | N | N | N | 2 | S | N | Yes | N | 2 |  | 6 |
| Evans et al 2002 | CBA | B | N | N | L | U | 1 | U | N | U | N | 0 | S | N | N | N | S | 2 | U | N | No | N | 0 |  | 3 |
| Blackman et al 2001 | UBA | C | N | S | S | N | 2 | N | N | U | N | 0 | L | S | N | N | N | 2 | L | N | Yes | N | 2 |  | 6 |
| Wells 2000 | UBA | C | N | L | S | N | 2 | N | N | U | N | 0 | L | N | N | N | N | 1 | L | N | Yes | N | 2 |  | 5 |
| Ambrose 1999 | UBA | C | N | S | S | N | 2 | N | N | U | S | 1 | S | S | N | N | S | 3 | S | N | Yes | N | 2 |  | 8 |
| Halpern 1995 | XUBA | C | N | S | S | N | 2 | N | N | U | S | 1 | L | S | N | N | N | 2 | N | N | Yes | N | 1 |  | 6 |
|  |  |  |  |  |  |  |  |  |  |  |  |  |  |  |  |  |  |  |  |  |  |  |  |  |  |
| **Mean**  **(range)** |  |  |  |  |  |  | 1.6  (0-2) | |  |  |  | 0.50 (0-1) |  |  |  |  |  | 2.40 (1-4) |  |  |  |  | 1.50 (0-2) |  | 6 (3-8) |
| **Intervention: Provision of basic housing needs/developing country intervention (n=6)** | | | | | | | | | | | | | | | | | | | | | | | | | |
| Cattaneo et al 2006 | RC | B | N | S | S | N | 2 | N | N | L | U | 1 | L | N | L | L | S | 4 | L | N | na | na | 1 |  | 8 |
| Choudhary et al 2002 | RC | B | N | N | S | N | 1 | N | N | N | S | 1 | S | L | S | N | N | 3 | S | N | na | na | 1 |  | 6 |
| Aga Khan Health Service 2001 | XCBA | B | N | N | S | N | 1 | N | N | N | L | 1 | S | L | N | N | N | 2 | S | N | na | na | 1 |  | 5 |
| Spiegel et al 2003 | XCBA | C | N | S | S | N | 2 | N | N | U | N | 0 | L | N | N | N | N | 1 | S | N | na | na | 1 |  | 4 |
| Aiga et al 2002 | XCBA | C | N | N | S | N | 1 | N | N | N | L | 1 | S | L | N | N | S | 3 | U | N | na | na | 0 |  | 5 |
| Wolff et al 2001 | XCBA | C | N | S | S | N | 2 | N | N | N | N | 0 | L | N | N | N | S | 2 | S | N | na | na | 1 |  | 5 |
|  |  |  |  |  |  |  |  |  |  |  |  |  |  |  |  |  |  |  |  |  |  |  |  |  |  |
| **Mean**  **(range)** |  |  |  |  |  |  | 1.50  (1-2) | |  |  |  | 0.67 (0-1) |  |  |  |  |  | 2.67 (1-4) |  |  |  |  | 0.83 (0-1) |  | 5.5 (4-8) |
|  |  |  |  |  |  |  |  |  |  |  |  |  |  |  |  |  |  |  |  |  |  |  |  |  |  |

**Web Table 2: Assessment codes for individual external validity items with domain score sub-totals (ST) and total score by study** *(continued)*

| **Intervention: Rehousing from slums (pre 1965) (n=4)** | | | | | | | | | | | | | | | | | | | | | | | | | |
| --- | --- | --- | --- | --- | --- | --- | --- | --- | --- | --- | --- | --- | --- | --- | --- | --- | --- | --- | --- | --- | --- | --- | --- | --- | --- |
| Wilner et al 1960 | CBA | A | N | S | S | N | 2 | N | N | N | S | 1 | S | S | N | S | N | 3 | S | N | Yes | N | 2 |  | 8 |
| McGonigle et al 1936 | XCBA | B | N | S | S | N | 2 | N | N | N | S | 1 | L | S | S | N | S | 4 | S | N | No | N | 1 |  | 8 |
| Ferguson 1954 | RC | C | N | N | L | N | 1 | N | N | U | N | 0 | L | N | N | N | N | 1 | S | N | na | na | 1 |  | 3 |
| Chapin 1938 | UBA | C | N | S | S | N | 2 | N | N | U | S | 1 | N | S | S | S | S | 4 | S | N | Yes | N | 2 |  | 9 |
|  |  |  |  |  |  |  |  |  |  |  |  |  |  |  |  |  |  |  |  |  |  |  |  |  |  |
| **Mean**  **(range)** |  |  |  |  |  |  | 1.75  (1-2) | |  |  |  | 0.75 (0-1) |  |  |  |  |  | 3.00 (2-4) |  |  |  |  | 1.50 (1-2) |  | 7.00 (3-9) |
|  |  |  |  |  |  |  |  | |  |  |  |  |  |  |  |  |  |  |  |  |  |  |  |  |  |
| **TOTAL (n=39 studies) MEAN**  **(range)** |  |  |  |  |  |  | 1.54  (0-2) | |  |  |  | 0.72 (0-2) |  |  |  |  |  | 2.49 (0-4) |  |  |  |  | 1.28 (0-2) |  | 6.00 (2-9) |

ST= sub-total; IVG= Internal validity grade (overall assessment of study quality)

Reporting of individual external validity items L= large extent; S= some extent; U= unclear; N= not at all; na= not applicable.

Study design: RCT: Randomised Controlled Trial; CBA: Controlled Before & After; UBA: Uncontrolled Before & After; XCBA: Cross-sectional Controlled Before & After; XUBA: Cross-sectional Uncontrolled Before & After; RC: Retrospective controlled; R: Retrospective uncontrolled.

List of references available in reference 4 (main manuscript text) or from the author
